# Genetic Epidemiology of Breast Cancer in Latin America

**DOI:** 10.3390/genes10020153

**Published:** 2019-02-18

**Authors:** Valentina A. Zavala, Silvia J. Serrano-Gomez, Julie Dutil, Laura Fejerman

**Affiliations:** 1 Department of Medicine, Division of General Internal Medicine, University of California San Francisco, San Francisco, CA 94143-1793, USA; valentina.zavala@ucsf.edu; 2 Grupo de investigación en biología del cáncer, Instituto Nacional de Cancerología, Bogotá 11001000, Colombia; silviajserrano@gmail.com; 3 Cancer Biology Division, Ponce Research Institute, Ponce Health Sciences University, Ponce, PR 00732, USA; jdutil@psm.edu

**Keywords:** genetic epidemiology, breast cancer, Latin America

## Abstract

The last 10 years witnessed an acceleration of our understanding of what genetic factors underpin the risk of breast cancer. Rare high- and moderate-penetrance variants such as those in the *BRCA* genes account for a small proportion of the familial risk of breast cancer. Low-penetrance alleles are expected to underlie the remaining heritability. By now, there are about 180 genetic polymorphisms that are associated with risk, most of them of modest effect. In combination, they can be used to identify women at the lowest or highest ends of the risk spectrum, which might lead to more efficient cancer prevention strategies. Most of these variants were discovered in populations of European descent. As a result, we might be failing to discover additional polymorphisms that could explain risk in other groups. This review highlights breast cancer genetic epidemiology studies conducted in Latin America, and summarizes the information that they provide, with special attention to similarities and differences with studies in other populations. It includes studies of common variants, as well as moderate- and high-penetrance variants. In addition, it addresses the gaps that need to be bridged in order to better understand breast cancer genetic risk in Latin America.

## 1. Introduction

Breast cancer is the most common cancer in women, and a major public health concern worldwide [1]. Improvements in screening, early detection, and treatment led to decreased mortality, a 12% decrease between 2002 and 2012 [2,3]. Breast cancer is a complex trait determined both by genetic and non-genetic factors and, in most cases, without a clear pattern of inheritance [4,5]. Twin studies have reported a heritability of 27–31% in Nordic populations [5,6,7], and recent analysis based on genome-wide association studies (GWAS) on individuals of American and European ancestry have reported a heritability between 9 to 13% [8,9].

Only a relatively small proportion of breast cancers can be explained by the presence of high-penetrance genetic mutations, such as those in the *BRCA1* and *BRCA2* genes [10,11,12,13,14,15,16,17,18,19]. These, together with mutations in genes of intermediate penetrance such as *ATM*, *BARD1*, *PALB2*, and *CHECK2*, explain ~20–25% of breast cancer risk [20], leaving a considerable proportion of heritability still to be explained by low-penetrance variants. Breast cancer GWAS were instrumental in the discovery of multiple single-nucleotide polymorphisms (SNPs), starting with relatively modest sample sizes in 2007 [21,22,23] and developing into major consortiums that now include more than 200,000 women [21,22,23,24,25,26,27,28,29,30,31,32,33,34,35,36,37,38,39,40,41,42,43,44,45,46,47,48,49,50,51]. These SNPs are not very informative on their own, but together may account for ~44% of the familial relative risk [34]. However, only a small proportion of samples included in these large efforts are from United States (US) minority populations (e.g., Hispanics/Latinas, African Americans).

Latin American populations are diverse, not only culturally, but also in their genetic ancestry composition [52,53]. This diversity is likely to have an impact on the distribution of genetic determinants of breast cancer risk in different regions. The aim of this review was to highlight breast cancer genetic epidemiology studies conducted in Latin America and to summarize the information that they provide, addressing gaps that need to be bridged in order to better understand breast cancer risk in Latin America.

For paper retrieval, we combined search terms such as “breast cancer” (breast cancer subtypes), “genetics” (genetic variant, genetic polymorphism, SNP, genetic mutation, *BRCA*), “case–control study” (cases/controls, cases–controls, cases, controls), and “Latinas” (Latinas, Hispanics, Latin America, South America) in the PubMed database (https://www.ncbi.nlm.nih.gov/pubmed/). We also added to these terms the name of each Latin American country. We complemented the search by reviewing manuscripts cited in the selected papers. We focused on studies with case series, cohorts, or case/control design, conducted in Latin America and that included genetic factors as main predictors. In some instances, we make reference to studies conducted in or including US Latinas. We only included studies published in the English language. We did not narrow the search by date; the selected studies were published between 2008 and 2018.

## 2. *BRCA1* and *BRCA2* Mutations in Latin America

The Hereditary Breast and Ovarian Cancer (HBOC) syndrome is caused by loss-of-function mutations in the *BRCA1* and *BRCA2* genes and explains approximately 16% of inherited breast cancers [13]. Despite being relatively rare, pathogenic variants in the *BRCA* genes are associated with substantial increases in risk of breast, ovarian, and other cancers [18,54,55,56,57,58,59,60]. The comparison of the prevalence of *BRCA1*/*BRCA2* pathogenic variants across studies in Latin America is confounded by variation in the study design, such as the approach for patient accrual, inclusion criteria, and screening technology. Nevertheless, available evidence points to marked differences in the carrier prevalence in different populations. Latin American countries, for which prevalence was assessed in unselected breast cancer patient cohorts, report a similar prevalence to that of non-Hispanic Whites (NHW) in the US; this includes Brazil [61], Colombia [62], Cuba [63], Mexico [64,65], Peru [66], and Puerto Rico [67], for which the prevalence in unselected breast cancer cases was shown to be below 5%. Differences in the relative proportion of *BRCA1* to *BRCA2* carriers were also observed. While most countries report higher frequencies of *BRCA1* mutations, in Costa Rica, Cuba, Puerto Rico, and Uruguay, *BRCA2* mutations are predominant [68]. This is expected to impact the clinical presentation of HBOC in those countries, as each gene is associated with differences in the age of onset, molecular profile, and the magnitude of risk spectrum of associated cancers [18,69].

### Founder Mutations and Regional Heterogeneity

Three founder mutations were shown to account for 79% of the *BRCA1*/*BRCA2* carriers in the Ashkenazi Jewish population [70]. In Latin America, these known founder Jewish mutations were reported to be recurrent (35% of mutation carriers) in a Jewish population of Buenos Aires, illustrating the immigration history of this country [71]. The *BRCA1* 185delAG (c.68_69delAG, rs80357914) is the most common pathogenic variant with the largest geographical span as it was shown to be recurrent in Argentina, Brazil, Mexico, and Peru [68,72]. In Mexican Americans, haplotype analysis demonstrated that this mutation was on the same genetic background as the Jewish founder mutation [73]. Founder mutations were also reported in Brazil, Chile, Colombia, Mexico, and more recently in Puerto Rico [67,72,74,75]. In addition to founder mutations, several countries reported the presence of recurrent mutations, but the data regarding a common origin or ancestor were not assessed [74]. It was estimated that less than 10% of the *BRCA* pathogenic variants reported in the literature were observed in more than one country of Latin America and the Caribbean [68,76,77]. In a large cohort distributed across 11 Brazilian states, some recurrent mutations were restricted to defined regions of the country, while other areas displayed significant heterogeneity [78]. In Colombia, despite a more modest sample size, a recent study reported a very low frequency of known Colombian founder mutations, which was associated with variations in geographic origin of the cohort within the country [79]. The genetic heterogeneity of Latin America populations suggests that further sequencing of patients in different countries and regions would be required to properly characterize geographical variation in the *BRCA* mutation spectrum and to provide adequate recommendations and treatment in women from Latin America. A previous review by Dutil et al. provided additional information regarding specific *BRCA* mutations in Latin American women [68]. In addition to mutations in the *BRCA* genes, there are other high-penetrance mutations that are uncommon in the population (e.g., *TP53*, *PTEN*, and *STK11*), which are associated with well-known familial syndromes [80,81,82]. For example, the c.1010G>A (rs121912664) variant in the *TP53* gene, best known as R337H, was found in 2–7% of Brazilian breast cancer patients [83,84,85].

## 3. Breast Cancer Candidate Gene Studies in Latin America

Polymorphisms in moderate- and low-penetrance genes may be relatively common in the population, and collectively confer a small to moderate increased relative risk for breast cancer [86]. Common polymorphisms located within genes in known cancer-related pathways are good candidates for investigation in the context of a case–control design and were explored in multiple Latin American studies (Table 1).

### 3.1. DNA Repair Genes

DNA repair is crucial for normal cell function, and common polymorphisms in DNA repair genes or genes that play a role in DNA repair pathways are potential candidates for association with breast cancer risk. Several studies in Latin America assessed the association between DNA repair gene variants and breast cancer risk [94,97,98,99,100,101,110,112,117,118,119,120,121,122,123]. Candidate genes that were most studied are those that code for proteins that interact with BRCA1/2 or that are involved in the same pathway. ATM is a protein kinase that finds double-strand breaks in DNA and controls the mechanism of DNA repair [124]. Three case–control studies assessed the effect of variants in the *ATM* gene in Chile and Mexico [99,100,112]. In both countries the variants c.3285-9delT (rs1799757), c.5497-8T>C (rs3092829), and c.5557G>A (rs1801516) were reported to be associated with breast cancer risk.

The BARD1 protein heterodimerizes with BRCA1 and induces translocation and retention of BRCA1 into the nucleus [125]. The c.1670G>C (rs28997576) variant was assessed in case–control studies from several populations [97,126,127,128,129,130,131,132]. In Chile, this variant was associated with an increased breast cancer risk in families with strong family history [97]. Two other variants were detected in a cohort of 124 Peruvian triple-negative breast cancer patients: the pathogenic c.334C>T (rs758972589) variant and the probably pathogenic c.1622C>A (rs777937955) variant [122].

Three other BRCA1 and BRCA2 functionally related genes were screened in Latin American populations. PALB2 binds BRCA2 and recruits it to BRCA1 foci on damaged DNA [133]. Leyton et al. screened this gene in 100 Chilean breast cancer patients negative for *BRCA1/2* mutations and found two variants, c.1676A>G (rs152451) and c.2993C>T (rs45551636), that were associated with breast cancer risk in a subsequent case–control study [94]. CHEK2 protein is activated by ATM and phosphorylates BRCA1 [134]. The *CHEK2* c.1100delC truncating mutation, which increases the risk of breast cancer twofold, was widely analyzed. This mutation has a frequency of 0.3–1.2% in European populations [135] and case-only studies in Brazil, and case–control studies in Colombia and Chile concluded that this variant is almost absent in these populations [119,123,136,137]. *AKT1* is frequently mutated in mammary tumors [138]. Activated AKT1 phosphorylates a large number of downstream substrates that play a crucial role in tumor growth and survival [139]. Lopez-Cortes et al. [102] analysed six SNPs (c.49G>A (rs121434592), c.956A>G (rs12881616), c.1070T>C(rs11555432), c.1162C>A (rs11555431), c.1172+23A>G (rs2494732), and c.1172+69G>C (rs3803304)) in *AKT1* and its association with breast cancer risk, as well as histopathological and immunohistochemical characteristics, in 276 Ecuadorian mestizo women. They found that the intronic SNP rs3803304 was associated with breast cancer risk. Moreover, they found that the heterozygous genotype CG of that SNP had a significantly different frequency between intrinsic subtypes (*p* ≤ 0.05), with luminal B showing the highest frequency (63.6%). This result suggests that the SNP rs3803304 may affect DNA transcription, acting as a risk factor for breast cancer in Ecuadorian mestizo population living in high altitudes.

Polymorphisms within two DNA double-strand break repair genes, *XRCC3* c.722C>T (rs861539) and *RAD51D* c.698A>G (rs28363284), were assessed for their association with breast cancer risk in high-risk families in Chile without known *BRCA1/2* mutations [98]. Only the variant in the *XRCC3* gene yielded a statistically significant result [98]. In relation to DNA single-strand break repair genes, a case–control study in Puerto Rico tested associations between SNPs in the xeroderma pigmentosum (*XPC* and *XPD*) and ultraviolet (UV) excision repair protein (*RAD23B*) genes and breast cancer risk [117]. They hypothesized that genetic variation in the nucleotide excision repair pathway genes could modulate DNA repair capacity and contribute to breast cancer risk and found an association between *RAD23B* and breast cancer risk, and between *XPC* and *RAD23B* and DNA repair capacity in the cases [117]. Similarly, a Mexican study reported a significant association between the c.1196A>G (rs25487) variant of the *XRCC1* gene and breast cancer risk [121]. Variants in *TP53*, which is an important gene in DNA-repair related pathways [140], were reported in Brazilian populations [83,84,85,114,141,142,143,144,145]. Four case-control studies assessed the rs1042522 (c.215C>A) variant, reporting negative results. The heterozygous frequency ranges between 40 and 60% and the frequency of the homozygous genotype is ~10%. These frequencies are also similar to the frequencies in the healthy population [141,142,143,144]. Even though the prevalence of this mutation is variable across different populations, these results are similar to the average frequencies observed worldwide [146]. Three other studies evaluated the c.1010G>A (rs121912664) variant, which was found in 2–7% of breast cancer patients and absent in controls [83,84,85].

### 3.2. Genes Involved in Amino-Acid and Nucleic-Acid Metabolism

The thymidylate synthase (*TS*) gene produces an enzyme that is involved in one-carbon metabolism and is crucial for DNA synthesis. A study in Mexico explored the association between polymorphisms in the *TS* gene and breast cancer risk and reported negative results [118]. Other case–control studies in Mexico, Ecuador, and Brazil [88,103,110,111,147] evaluated the association between polymorphisms in the methylenetetrahydrofolate reductase (*MTHFR*) and cystathionine β synthase (*CBS*) genes, which are involved in the processing of amino acids, and breast cancer risk. Four out of five studies found a positive association [88,103,110]. López-Cortés et al. [103] did not find statistically significant differences between breast cancer subtypes but reported an association between the heterozygous C/T and homozygous mutant T/T genotypes for the c.665C>T (rs1801133) polymorphism, and a lower *MTHFR* messenger RNA (mRNA) expression in triple-negative breast cancer (TNBC) tumors. The 5-methyltetrahydrofolate-homocysteine methyltransferase (*MTR*) gene, also involved in the amino-acid synthesis pathway, was tested in a Brazilian population with no significant results [147].

### 3.3. Genes Involved in Hormone Metabolism, and Hormone Receptor, Co-Activator, or Suppressor Genes

Sex hormones, their receptors, and other relevant components within their pathways, are thought to be involved in breast cancer development because of their role in cell growth and proliferation. Analyses of a limited number of candidate variants in hormone receptor genes (estrogen (*ESR1*), androgen (*AR*), and progesterone receptors (*PGR*)) yielded mostly negative results except in secondary stratified analyses (by body mass index or use of hormone replacement therapy) [148,149,150,151,152]. Studies conducted in Latin America focused on the *ESR1*, *PGR*, *COMT, CYP19A1*, and *CYP1A1* genes [89,113,115,120,150,153]. One of these studies reported a positive association between an Alu insertion in the *PGR* gene and breast cancer risk [120], while other studies revealed statistically significant associations for polymorphisms within the *CYP1A1* gene [113,115].

### 3.4. Extracellular Matrix Components 

Matrix metalloproteinases (MMPs) are enzymes that can degrade the extracellular matrix and basement membrane and are thought to play an important role in cell proliferation, migration, differentiation, apoptosis, and angiogenesis [154]. A large study including US Hispanic/Latina women tested associations between polymorphisms in *MMP-1*, *MMP-2*, *MMP-3*, and *MMP-9* genes and breast cancer risk [155]. Significant associations were found for intronic SNPs in the *MMP-1* (c.625+332G>A, rs996999), *MMP-3* (c.1229+495C>T, rs650108), and *MMP-9* (c.1331-163G>T, rs3787268) genes among women with high Native-American ancestry [155]. A study conducted in Mexico reported a strong association between the *MMP-2* c.-1586C>T polymorphism (rs243865) and breast cancer risk [156]. Other studies in Argentinian and Brazilian populations reported negative results for two SNPs (c.781G>A, rs11895564 and c.3841C>T, rs121912467) in the integrin subunit α6 (*ITGA6*) gene and the c.-1562 C>T (rs3918242) polymorphism in the *MMP-9* gene, respectively [157,158].

### 3.5. Inflammation and Energy Balance

Different analyses conducted in the Breast Cancer Health Disparities Study samples analyzed tag SNPs in genes related to inflammation and energy balance, taking into account possible interactions with body mass index [159,160,161,162,163,164]. Few Latin American studies tested the association between genes in inflammation and energy balance pathways and breast cancer risk (*TNF-α*, *RETN*, *CAP1*, and *PTGS2* genes). No association was detected for nine SNPs evaluated within the *PTGS2* gene [165]. The *TNF-α* c.-308G>A (rs1800629) polymorphism was found to be associated with breast cancer risk in a study conducted in Mexico [166], and another Mexican study found associations between the *RETN* c.-225C>G (rs1862513) and *CAP1* c.881G>A (rs35749351) polymorphisms and breast cancer risk [104].

### 3.6. Genes Associated with Tumoral Immunity

Two studies assessed polymorphisms related to tumor immune response in Brazilian populations, and both of them found statistically significant associations with breast cancer risk [87,90]. Tumor-infiltrating regulatory T cells (Tregs) possess relevant roles in tumor immunity. When activated by tumor-associated antigens, they can suppress specific antitumor immune responses [167]. Forkhead box P3 (FOXP3) is a transcription factor essential for the development and functions of Treg cells [87,168]. It was also reported as being expressed in breast cancer cells, and its expression is associated with a better prognosis after neoadjuvant chemotherapy in *HER2*-overexpressing breast cancer patients [168]. Banin Hirata et al. (2017) analyzed two FOXP3 SNPs (c.-22-902A>G (rs2232365) and c.-23+2882C>A (rs3761548)) and their possible association with susceptibility and clinical outcomes. They reported associations between genotypes and clinical characteristics for specific breast cancer subtypes [87], suggesting that FOXP3 may influence clinical outcome according to intrinsic subtype and might be a marker for aggressive disease. Another study reported associations between two SNPs in the promoter of the *IL-18* gene, c.-838C>A(rs1946518) and c.-368G>T (rs187238), and breast cancer risk [90]. IL-18 is a pleiotropic cytokine that promotes antitumor pro-inflammatory responses and is highly expressed in breast cancer tumors in mice and in serum of breast cancer patients [169,170]. Predictive models showed that these SNPs might impair the binding of some transcription factors, decreasing promoter activity [171,172].

### 3.7. Metabolism of Xenobiotic Compounds and Oxidative Stress

Selenoproteins were shown to have a redox function and to decrease oxidative stress. One study tested the association between SNPs in different selenoprotein-related genes (*GPX1, GPX2, GPX3, GPX4, SELS, SEP15, SEPN1, SEPP1, SEPW1, TXNRD1*, and *TXNRD2*) and breast cancer risk in US Latinas, finding mostly negative results except when analyses were stratified by genetic ancestry and by tumor subtype [173]. Other studies were conducted in Mexico and Brazil and investigated multiple genes: *CYP2W1, CYP4F11*, *CYP8A1*, and *CYP1B1* [174], which are important in the activation of carcinogenic compounds; *ABCB1*, which encodes the MDR1 protein, [105,106] and is involved in the elimination of xenotoxic agents; *GSTM1* and *GSTP1*, members of the glutathione *S*-transferase (*GST*) gene family [91,108,109,113]; and *eNOS*, which can react with other free radicals and damage DNA [114]. Results for the *CYP* genes were mostly negative, except for *CYP1B1* [107], while positive associations were reported for the *GSTM1* deletion (tagged by rs366631), and *ABCB1* c.3435C>T (rs1045642), *GSTP1* c.313A>G (rs1695), and *eNOS* polymorphisms [91,105,106,108,109,113,114,174]. A study that included 275 Mexican women enrolled in the Ella Binational Breast Cancer Study reported that the T allele of the c.3435C>T polymorphism located in exon 26 of the *ABCB1* gene was an important risk factor for breast cancer in premenopausal women and specifically for the TNBC subtype [106]. This polymorphism does not change the amino-acid sequence, but is associated with a decrease in *ABCB1* function, and reduced mRNA and/or protein expression, which might impact the metabolism and posterior elimination of toxic and carcinogenic compounds inducing cellular accumulation leading to cancer development [106,175].

### 3.8. Non-Coding RNAs

Non-coding RNAs participate in complex networks involved in cancer pathways. MicroRNAs are short non-coding RNAs with relevant roles in the physiological control of cell homeostasis as they regulate the expression of hundreds of genes though direct binding to their mRNAs [176]. SNPs on microRNA precursors (pre-microRNAs) can influence the processing into their functional mature forms; moreover, SNPs in the mature forms may affect the strength of regulation of their target mRNAs. Two studies tested the association of SNPs within microRNAs in Latina patients [92,93]. The first study assessed the effect of the miR-196a2 rs11614913 SNP in a Brazilian population and revealed that the T allele was associated with increased breast cancer risk, while the CC genotype had a protective effect [92]. In a Chilean study, SNPs in pre-miR-27a, pre-miR-196a2, pre-miR-423, pre-miR-618, and miR-608 were tested for their association with breast cancer risk [93]. The authors found that the pre-miR-423 rs6505162 and pre-miR27a rs895819 SNPs were associated with breast cancer risk in individuals with a strong family history and with a moderate history of breast cancer, respectively, and that the pre-mir-618 rs2682818 was associated with an increased risk in non-familial early-onset breast cancer [93].

## 4. Genome-Wide Association Studies in Latin America

The first GWAS on Latina women was published in 2014 [40], reporting genome-wide statistically significant results for two linked SNPs 56 kb upstream of the *ESR1* gene (rs140068132 and rs147157845). These SNPs have a frequency of between 5% and 23% in Latin American populations and are practically absent in all other groups. The minor allele was protective, with an associated odds ratio (OR) of 0.60 (95% confidence interval (CI): 0.53–0.67) and was more protective for estrogen-receptor-negative (ER^−^) (OR: 0.34; 95% CI: 0.21–0.54) than for estrogen-receptor-positive disease (OR: 0.63; 95% CI: 0.49-0.80) [40]. An additional GWAs and fine mapping analysis of the 6q25 region in an extended sample identified three new SNPs associated with breast cancer risk [177].

Some studies conducted in Latin America replicated selected loci that were discovered in breast cancer GWAS conducted in samples of European origin: two in Chile and one in Mexico [95,96,116]. These studies tested the association between SNPs in *FGFR2*, *TOX3*, and *MAP3K1* genes and the 2q35 and 8q24 loci, that were replicated in different populations [178,179,180,181,182,183,184]. FGFR2 and MAP3K1 are tyrosine kinases. FGFR2 mediates signaling of fibroblast growth factors, MAP3K1 is involved in apoptosis signaling, and TOX3 is a nuclear protein that acts as a chromatin remodeler. Altered expression of these proteins was described in breast cancer pathogenesis [185,186,187]. Two studies assessed the association between SNPs in *FGFR2* (c.109+906T>C (rs2981582), c.109+1899A>G (rs2420946), 109+7033T>A (rs1219648), *MAP3K1* (rs889312), *TOX3* (rs3803662), 2q35 (rs13387042), and 8q24 (rs13281615) and breast cancer risk in Chilean patients [95,96]. They found associations for SNPs on *FGFR2*, *MAP3K1*, *TOX3*, and 2q35, but not for 8q24 [95,96]. Another analysis of the *FGFR2* rs2981582 polymorphism in the Mexican population reported an interaction between the *FGFR2* polymorphism and alcohol intake [116]. The first breast cancer GWAS in US Hispanic/Latinas also replicated previous associations, with most of the SNPs being concordant in terms of direction and magnitude of association with those reported in Europeans or Asians [40].

## 5. Genetic Ancestry and Breast Cancer Risk

There is a heterogeneous genetic background in Latin American populations, with regional variation in the relative proportions of the three main ancestral continental influences: Indigenous American, European, and African. A study conducted in US Hispanic/Latinas from the Bay Area first reported the association between genetic ancestry and breast cancer risk [188]. Women with higher European ancestry had an increased risk of breast cancer compared to women with higher Indigenous American ancestry. Subsequent studies in Latin American populations (two in Mexicans and one in Colombians) replicated this finding [189,190,191]. A study from Uruguay did not find a statistically significant difference in genetic ancestry between breast cancer cases and controls, possibly due to the lack of statistical power given the size of the sample and the relatively small proportion of Indigenous American ancestry in this population [192]. The original finding that genetic ancestry was associated with breast cancer risk in Latinas was followed by an admixture mapping [193] analysis and a breast cancer GWAS [40], which we previously mentioned, and led to the discovery of a protective genetic variant near the *ESR1* gene mostly observed in women of Indigenous American ancestry.

A large proportion of the case/control studies described in Section 3 did not adjust for genetic ancestry, which is a known confounder in genetic epidemiology studies. Self-reported ethnicity is a useful way to control for population stratification on an ethnicity-matched case–control study design from comparable geographical regions [194]; however, this methodology is logistically challenging and costly. In addition, given the complex genetic substructure of Latin American populations, it is highly recommended to adjust for covariates that capture the ancestry composition of cases and controls using panels of ancestry informative markers or genome-wide genotype data [195].

## 6. Conclusions and Perspectives

In this review, we presented a summary of genetic association studies in Latin American women, including studies of relatively rare high-penetrance mutations and those discovered using candidate gene approaches or GWAS, which tend to be of moderate or small effects but are more common.

In recent years, a picture of the *BRCA1*- and *BRCA2*-associated hereditary breast cancers in Latin America emerged. While still incomplete, available evidence points to a unique genetic make-up characterized by remarkable diversity across and within countries [68,74,75,196]. Yet, a better understanding of the prevalence and types of mutations underlying hereditary breast cancers in Latin American women is a necessary step for informing the strategies for the detection and clinical management of carriers. The use of affordable targeted mutation panels presents an attractive solution in settings with limited resources, which are common in many Latin American countries, provided that larger studies representative of the population of interest are conducted during variant discovery. These populations also present unique opportunities to contribute to the classification of variants of unknown significance. In recent years, clinical testing for hereditary breast cancer transitioned from *BRCA1* and *BRCA2* analysis to extended panels of genes including high- and moderate-penetrance risk genes. Although this approach became standard of care in the US, the data on the use of these panels in Latin American populations are sparse. In parallel to emerging efforts aimed at describing the molecular characteristics of hereditary breast cancers in Latin America, there is a need to improve access to genetic testing and for the development of supporting infrastructures in cancer risk assessment, education, and genetic counseling [197,198].

Risk prediction models that combine non-genetic and genetic risk factors were developed and are constantly being improved [199,200,201,202]. Taken separately, variants of small effect sizes have limited impact on breast cancer risk prediction, but when combined into polygenic risk scores (PRS), the magnitude of their effect on risk may equal or surpass those of pathogenic variants in moderate-risk genes [203]. Prediction models that are usually applied are based on research conducted in European populations [204]. Because GWAS SNPs are selected for their tagging properties rather than their potential functional significance, known differences in linkage disequilibrium structures in populations of the world [205] are likely to confound replication studies and the clinical validity of the European GWAS SNPs. Recently, Wang et. al. demonstrated that 30–40% of the SNPs commonly used to estimate breast cancer PRS have inconsistent directionality in African American populations and, consequently, performed poorly in this group [206].

Progress in the discovery of population-specific breast cancer genetic risk variants in this region is limited by different factors, such as the slower incorporation of new genotyping and sequencing technologies, and the relatively small samples sizes partly due to the paucity of National Cancer Registries and the inconsistent collection of biospecimens (i.e., tumor blocks) for research (Table 2). On average, approximately half of the genome of Latina American women is of European origin and, therefore, much larger sample sizes would be required if similar results as for the European genome are to be expected for the Indigenous American component. We cannot assume that only overlapping variants between different ancestral genomes will be associated with risk and, therefore, we need to focus our efforts to reduce research disparities by expanding available resources to include large cohorts and case–control studies of diverse populations in and outside the US.

There are ongoing efforts to improve homogeneity of study design and increase sample size in studies in Latin America. In 2009, the Center for Global Health of the US National Cancer Institute (NCI) created the US-Latin America Cancer Research Network (LACRN), a collaborative management structure that includes Argentina, Brazil, Chile, Mexico, and Uruguay [207]. In 2011 the US–LACRN launched the “Molecular Profiling of Stage II and III Breast Cancer in Latin American Women Receiving Standard of Care Treatment (MPBC)” study. Future studies will involve genetic ancestry estimation, sequencing of high-penetrance genes, whole-exome sequencing, and identification of circulating tumor cells and cell-free DNA. The Molecular Subtypes of Premenopausal Breast Cancer in Latin American Women (PRECAMA) study is an effort led by the International Agency for Research on Cancer (IARC) involving data harmonization and standard protocols for recruitment of premenopausal breast cancer cases and controls, collection and storage of blood samples, tumor block fixation and handling, and pathology review in Chile, Colombia, Mexico, and Costa Rica [208]. Similarly, the EPIGEN-Brazil Initiative aims to study the association between genetic variants found in the Brazilian population and complex diseases, taking into account its complex admixture genetic background [209]. Many studies in Latin America are based on the recruitment of cases only and, therefore, cannot be included in larger studies based on case–control comparisons. However, efforts such as the Consortium for the Analysis of the Diversity and Evolution of Latin America, the Candela project, which aims to characterize the physical appearance and to examine the genetic and social background of 7342 subjects from Mexico, Colombia, Peru, Chile, and Brazil [210], could be leveraged to complement case-only designs. Another such resource is the Chile Genomico Project, aimed at describing the genomic structure of the Chilean population based on the contribution of the Indigenous Americans, European, and African populations. So far, they have genotyped 3500 individuals (chilegenomico.cl; Accessed February 10th 2019). Finally, the Mexico Biobank Project has created a nation-wide DNA biobank that facilitates research on complex diseases and aims to characterize the population genetics of native and admixed Mexicans (mxbiobankproject.org; Accessed February 10th, 2019).

To conclude, large collaborative international efforts that include knowledge and technology exchange and transfer will lead to studies with appropriate sample sizes and approaches to discover rare high-penetrance polymorphisms, as well as low-penetrance variants in Latin American women from different regions and with different ancestral proportions. Only then will we be able to provide appropriate genetic risk estimates and care to women of Latin American origin worldwide. 

## Figures and Tables

**Table 1:** Summary results from breast cancer candidate gene studies in Latin America showing a positive association with cancer risk (*p* ≤ 0.05).

Country	Gene	Variant	Risk Genotype vs. Common Genotype	OR (95% CIs) *p*-Value	Model	Design	Reference
	*FOXP3*	rs2232365A>G	AA vs. GG	1.93 (1.01–3.66) *p* = 0 046	Controlled by age	117 cases and 300 controls	Banin Hirata et al., 2017 [87]
Brazil	*MTHFR*	rs1801133C>T	TT vs. (CC + CT)	2.53 (1.08–5.93) *p* = 0.03	Adjusted for age, alcohol and smoking consumption, and BMI	100 cases and 144 controls	Zara-Lopes et al., 2016 [88]
	*CYP1A1*	rs1048943A>G	(AG + GG) vs. AA	1.50 (1.14–1.97) *p* = 0.004	Adjusted for age and ethnic origin	742 cases and 742 controls	Oliveira et al., 2015 [89]
	*IL-18*	rs1946518C>A	CC vs AA	2.78 (1.39–5.59) *p* = 0.004	Adjusted for age and BMI	154 cases and 118 controls	Back et al., 2014 [90]
		rs187238G>C	(GG + GC) vs. CC	3.89 (1.43–10.64) *p* = 0.008			
	*GSTM1*	rs366631C>T	−/− vs. (+/− and +/+)	2.40 (1.1–5.6) *p* =0.04	Not adjusted	49 cases and 49 controls	Possuelo et al., 2013 [91]
	*miR-196a2*	rs11614913C>T	(TC+ TT) vs. CC	1.50 (CI not provided) *p* = 0.014	Adjusted for age, race, menarche age, menopausal status, smoking habits, and first-degree breast cancer family history	388 cases and 388 controls	Linhares et al., 2012 [92]
Chile	pre-mir-423	rs6505162C>A	AA vs. CC	1.70 (1.0–2.0) *p* = 0.05	In families with at least three BC and/or OC cases	440 cases ^a^ and 807 controls	Morales at al., 2016 [93]
	pre-mir-618	rs2682818C>A	CA vs. CC	1.60 (1.0 − 2.4) *p* = 0.04	In families with a single case, diagnosed at ≤ 50 years of age		
	pre-mir-27a	rs895819A>G	GG vs. AA	0.30 (0.1–0.8) *p* = 0.01	In families with 2 BC and/or OC cases		
	*PALB2*	rs152451A>G	AG vs. AA	2.0 (1.2–3.1) *p* < 0.01	In families with at least three BC and/or OC cases	436 cases ^a^ and 809 controls	Leyton et al., 2015 [94]
		rs45551636C>T	CT vs. CC	3.0 (1.2–6.8) *p* < 0.01			
	*TOX3*	rs3803662C>T	TT vs. CC	2.38 (1.44–3.90) *p* = 0.0003	In families with at least 2 BC and/or OC cases	344 cases ^a^ and 801 controls	Elematore et al., 2014 [95]
	2q35	rs13387042 G>A	AA vs. GG	1.99 (1.25–3.14) *p* = 0.0015			
	*FGFR2*	rs2981582C>T	CT vs. CC	2.00 (1.26–3.23) *p* = 0.002	In families with a single case, diagnosed at ≤ 50 years of age	351 cases ^a^ and 802 controls	Jara et al., 2013 [96]
		rs2420946C>T	TT vs. CC	2.05 (1.16–3.63) *p* = 0.009			
		rs1219648A>G	GG vs. AA	2.06 (1.16–3.66)*p* = 0.011			
	*MAP3K1*	rs889312A>C	CC vs. AA	1.96 (1.13–3.37) *p* = 0.016			
	*BARD1*	rs28997576C>G	CG vs.CC	3.4 (1.2–10.2) *p* = 0.04	In families with at least three BC and/or OC cases	322 cases ^a^ and 570 controls	Gonzalez-Hormazabal et al., 2012 [97]
	*XRCC3*	rs861539C>T	TT vs. CC	3.2 (1.40–1.72) *p* = 0.006	In families with at least three BC and/or OC cases		
			TT vs. CC	2.44 (1.34–4.43) *p* = 0.003	Not adjusted	267 cases ^a^ and 500 controls	Jara et al., 2010 [98]
	*ATM*	rs1801516G>A	(GA + AA) vs. GG	2.52 (1.33–4.77) *p* = 0.008	Not adjusted	42 cases ^a^ and 200 controls	Tapia et al., 2008 [99]
		rs1801516G>A	GA vs. GG	1.74 (0.96–3.16) *p* = 0.048	Not adjusted	126 cases ^a^ and 200 controls	Gonzalez-Hormazabal et al., 2008 [100]
		IVS24-9delT	T(-T) vs. TT	1.74 (0.96–3.16) *p* = 0.048			
		IVS38-8T>C	TC vs. TT	3.09 (1.11–8.59) *p* = 0.024			
	*RAD51*	rs1801320G>C	(GC + CC) vs. GG	2.17 (1.11–4.24)*p* = 0.020	In patients <50 years at diagnosis	143 cases ^a^ and 247 controls	Jara et al., 2007 [101]
Ecuador	*AKT1*	rs3803304C>G	GG vs. CC	5.20 (1.3–20.9) *p* ≤ 0.05	Not adjusted	91 cases 185 controls	Lopez-Cortes et al., 2018 [102]
	*MTHFR*	rs1801133C>T	TT vs. CC	2.9 (1.2–7.2) *p* = 0.025	Not adjusted	114 cases and 195 controls	Lopez-Cortes et al., 2015 [103]
Mexico	*RETN*	rs1862513C>G	(CG + GG) vs. CC	1.62 (1.025–2.557) *p* = 0.03791	Not adjusted	100 cases and 308 controls	Muñoz-Palomeque et al., 2018 [104]
	*CAP1*	rs35749351G>A	(GA + AA) vs. GG	2.19 (1.075–4.475) *p* = 0.0277	Not adjusted		
	*ABCB1*	rs1045642C>T	CC vs. TT	2.91 (1.48–5.74) *p* = 0.001	Not adjusted	243 cases and 118 controls	Jaramillo-Rangel et al., 2018 [105]
			CT vs. CC	2.27 (1.11–4.67) *p* = 0.023	In premenopausal women	248 cases and 180 controls	Gutierrez-Rubio et al., 2015 [106]
	*CYP1B1*	rs1056827G>T	TT vs. GG	1.21 (0.85–1.72) *p* = 0.04	Adjusted for age, years of education, first-degree relative with breast cancer, age at first full-term pregnancy, breastfeeding at first birth, consumption of alcohol and tobacco, and genetic ancestry (Native American, European, and African).	952 cases and 998 controls *	Garcia-Martinez et al., 2017 [107]
	*GSTM1*	rs366631C>T	TT vs. (CC + CT)	1.30 (1.02–1.8) *p* = 0.04	Not adjusted	558 cases and 276 controls	Soto-Quintana et al., 2015 [108]
			TT vs. (CC + CT)	2.19 (1.50–3.21) *p* = 0.001	Not adjusted	243 cases and 118 controls	Jaramillo-Rangel et al., 2015 [109]
	*MTHFR*	rs1801133C>T	TT vs. CC	2.50 (1.6–3.8) *p* = 0.0001	Not adjusted	497 cases and 339 controls	Ramos-Silva et al., 2015 [110]
	*CBS*	844ins68	(−/+) vs. (−/−)	2.2 (1.5–3.3) *p* = 0.0001	Not adjusted	323 cases and 371 controls	Gallegos-Arreola et al., 2014 [111]
	*ATM*	IVS24-9delT	T(-T) vs. TT	3.02 (1.24–7.30) *p* = 0.0122	Not adjusted	94 cases and 97 controls	Calderón-Zúñiga et al., 2014 [112]
	*GSTP1*	rs1695A>G	GG vs. AA	3.28 (1.44–7.50) *p* = 0.005	In premenopausal women	150 cases and 382 controls	Martinez-Ramirez et al., 2013 [113]
	*eNOS*	4a/b polymorphism	ab vs. bb	2.0 (1.3–3.1) *p* = 0.001	Not adjusted	429 cases and 281 controls	Ramírez-Patiño et al., 2013 [114]
	*CYP1A1*	rs1048943A>G	GG vs. AA	2.77 (1.30–5.89) *p* = 0.009	In premenopausal women	150 cases and 382 controls	Martinez-Ramirez et al., 2013 [113]
		rs4646903T>C	CC vs. TT	3.38 (1.05–10.87) *p* = 0.041	In postmenopausal women	91 cases and 94 controls	Moreno-Galvan et al., 2010 [115]
	*FGFR2*	rs2981582C>T	TT vs. CC	1.69 (1.25–2.27) *p* = 0.001	Adjusted by design for place of residence, health service institution membership, and 5-year age interval	687 cases and 907 controls *	Murillo-Zamora et al., 2013 [116]
Puerto Rico	*RAD23B*	rs1805329C>T	TT vs. (CT + CC)	3.14 (1.65–5.97) *p* < 0.001	Adjusted for age, civil status, education level, and contraceptive use	228 cases and 418 controls	Perez-Mayoral et al., 2013 [117]

CI: confidence interval; BC: breast cancer; BMI: body mass index; OC: ovarian cancer; OR: odds ratio. ^a^ Patients were tested for *BRCA1/2* mutations. * Population-based controls.

**Table 2:** Breast cancer genetic epidemiology in women of Latin American origin: Current limitations and possible solutions.

Current Limitations	Possible Solutions
Diverse genetic backgrounds and mutational frequencies among Latin American populations.	Foster collaborations between Latin American countries. Account and stratify for ancestry proportions.
Small sample size in comparison with studies including European and European American individuals.	Promote access and exchange of information among researches to establish research partnerships within and across countries to generate large consortiums for joint data analysis. Homogenize the design and data processing of studies from different countries to facilitate data sharing and sample pooling. Extension of National Cancer Registries and quality improvement, including biospecimen collection.
Limited access to high-cost technologies for variant discovery in multiple Latin American countries.	Promote collaborative relationships with specialized multicenter initiatives to reduce costs and assure quality control. Promote collaborations that allow Latin American countries to access technologies in the United States and other countries (e.g., European countries, Australia, Japan, etc.) with appropriate data sharing protocols that allow analyses to be conducted by scientists in Latin America.
Inequality in access to healthcare by genetic ancestry might limit the representation of highly Indigenous American women in genetic studies.	Foster active role of public hospitals in patient accrual. Decentralization of institutions in charge of patient/individual accrual.
